# Retraction: p63 and p73 Transcriptionally Regulate Genes Involved in DNA Repair

**DOI:** 10.1371/journal.pgen.1010699

**Published:** 2023-03-27

**Authors:** The PLOS Genetics Editors

Following the publication of this article [1], concerns were raised regarding Fig 5. Specifically,

- The following data appear more similar than would be expected from experiments representing different conditions:
  ○ RAD51-2&3, lane 6 (WT p73 D) and lane 18 (p63-/- p73 D).
  ○ mre11-3, lane 4 (WT p63 D) and lane 10 (p53-/- p63 D).
  ○ Input, lanes 1–12 and lanes 13–24, flipped horizontally and resized.
- When brightness/contrast levels are adjusted, there appear to be discontinuities suggestive of splice lines in the following locations:
  ○ BRCA2 panel, horizontally near the bottom of the panel from lanes 1 to 16 (WT p53 U to p63-/- p63 D), vertically between lanes 16 & 17 (p63-/- p63 D and p63-/- p73 U), and on the upper edge of the panel between lanes 22 & 23 (p73-/- p63 D and p73-/- p73 U).
  ○ Mre11-3 panel, between lanes 13 & 14 (p63-/- p53 D and p63-/- p63 U) and between lanes 18 & 19 (p63-/- p73 D and p73-/- p53 U).

The corresponding author stated that the original uncropped images underlying Fig 5 are no longer available. They stated that, to their knowledge, the images were not manipulated.

In light of the concerns affecting multiple figure panels that question the integrity of these data, the *PLOS Genetics* Editors retract this article.

SS, KG, GWB, and TJ agreed with the retraction. ERF did not agree with the retraction. YLL either did not respond directly or could not be reached. SS, KG, and GWB stand by the article’s findings. KG apologizes for the issues with the published article.

## References

[1] Lin Y-L, Sengupta S, Gurdziel K, Bell GW, Jacks T, Flores ER (2009) p63 and p73 Transcriptionally Regulate Genes Involved in DNA Repair. PLoS Genet 5(10): e1000680. 10.1371/journal.pgen.1000680 19816568 PMC2752189

